# Knee orthopedics as a template for the temporomandibular joint

**DOI:** 10.1016/j.xcrm.2021.100241

**Published:** 2021-04-14

**Authors:** Benjamin J. Bielajew, Ryan P. Donahue, M. Gabriela Espinosa, Boaz Arzi, Dean Wang, David C. Hatcher, Nikolaos K. Paschos, Mark E.K. Wong, Jerry C. Hu, Kyriacos A. Athanasiou

**Affiliations:** 1 Department of Biomedical Engineering, University of California, Irvine, Irvine, CA, USA; 2 Department of Surgical and Radiological Sciences, School of Veterinary Medicine, University of California, Davis, Davis, CA, USA; 3 Department of Orthopaedic Surgery, University of California, Irvine Medical Center, Orange, CA, USA; 4 Diagnostic Digital Imaging Center, Sacramento, CA, USA; 5 Department of Orthopaedic Surgery, Massachusetts General Hospital, Harvard Medical School, Boston, MA, USA; 6 Department of Oral and Maxillofacial Surgery, University of Texas School of Dentistry, Houston, TX, USA

**Keywords:** temporomandibular joint, knee joint, cartilage, TMJ disc, temporomandibular disorder, osteoarthritis, oral and maxillofacial surgery, orthopedic surgery, tissue engineering, translational medicine

## Abstract

Although the knee joint and temporomandibular joint (TMJ) experience similar incidence of cartilage ailments, the knee orthopedics field has greater funding and more effective end-stage treatment options. Translational research has resulted in the development of tissue-engineered products for knee cartilage repair, but the same is not true for TMJ cartilages. Here, we examine the anatomy and pathology of the joints, compare current treatments and products for cartilage afflictions, and explore ways to accelerate the TMJ field. We examine disparities, such as a 6-fold higher article count and 2,000-fold higher total joint replacement frequency in the knee compared to the TMJ, despite similarities in osteoarthritis incidence. Using knee orthopedics as a template, basic and translational research will drive the development and implementation of clinical products for the TMJ. With more funding opportunities, training programs, and federal guidance, millions of people afflicted with TMJ disorders could benefit from novel, life-changing therapeutics.

## Graphical abstract

**Figure:**
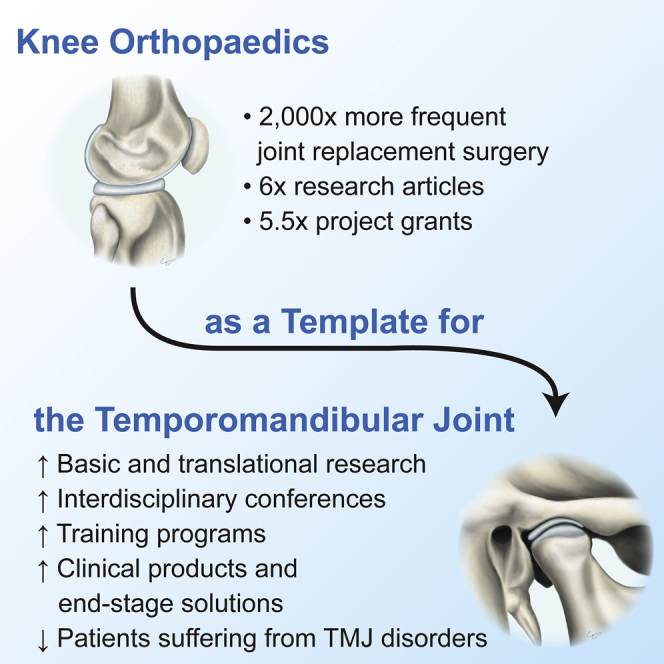

## Teaser

In this review, Bielajew et al. describe the stark quantitative differences in the knee orthopedics and temporomandibular joint (TMJ) fields, despite similarities in disease incidence. The authors call on researchers and clinicians to use knee orthopedics as a template to improve product development and end-stage outcomes for people with TMJ disorders.

## Introduction

**Figure 1:**
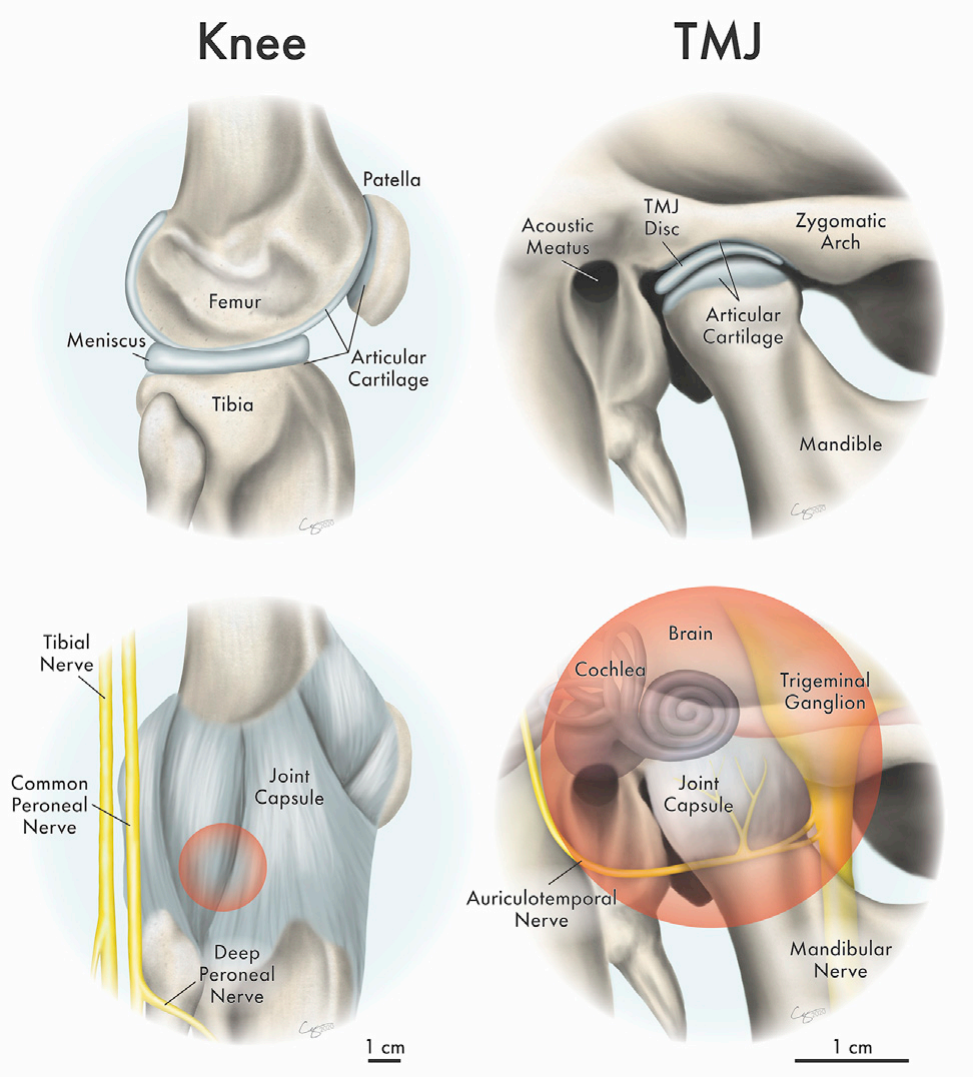
Knee and TMJ anatomy and proximity to crucial sensory structures (Top) Both the knee and TMJ are diarthrodial joints with two articular surfaces and an interpositional fibrocartilage. Specifically, the meniscus is situated between the tibia and the femur in the knee, and the TMJ disc is situated between the zygomatic arch and the mandible. (Bottom) Within a 3-cm sphere (red circle representation in 2D space) centered around the meniscus and the TMJ disc, the knee has no crucial sensory structures, although the TMJ has numerous structures present, including components of the inner ear, the brain, the trigeminal ganglion, and the mandibular and auriculotemporal nerves.

The knee joint and temporomandibular joint (TMJ) are two of the most used joints in the body. They are both diarthrodial hinge joints consisting of a fibrocartilaginous meniscus/disc between two articulating surfaces (Figure 1).^1^^,^^2^ The knee is better described as two joints, the tibiofemoral joint and the patellofemoral joint; these work together for flexion, extension, and rotation of the lower legs. The knee is the largest joint in the body and is essential for walking, running, and jumping.^3^ The TMJ is one of the most complex joints in the body and functions in rotation and translation to perform crucial activities, such as chewing, speaking, and breathing.^4^ Both joints are critical for performing many day-to-day movements, where they withstand large, repeated forces.

Although there are analogous structures in the knee and TMJ (Figure 1), there are some biomechanical and biochemical differences between the two joints. When performing simple motions, the knee withstands comparatively large forces; light jogging can put over four times the body’s weight (e.g., 3,080–3,600 N) on the knee^5^^,^^6^ compared to the TMJ, which experiences forces equivalent to the body’s weight (e.g., 770–900 N) when biting.^5^^,^^7^ Although compression and shear are major loading types in both joints,^8^ tensile loading plays a greater role in the TMJ than the knee.^9^ The knee meniscus contains zonal differences in collagen type I and II ratios, although the TMJ disc is almost completely composed of collagen type I.^10^ Although both articular surfaces contain growth plates, the mandibular condyle contains a unique fibrous zone,^11^ unlike the articular cartilages of the knee, which are completely hyaline. Despite these differences, the two joints manifest pathologies and disorders, leading to pain and dysfunction.

Approximately 25% of adults have some sort of cartilage affliction.^12^ Arthritides, diseases involving joint inflammation and cartilage degeneration, frequently occur from overuse, aging, trauma, or pathology. Osteoarthritis (OA) is the most common form, with the knee being one of the most frequently affected joints; about 14% of the adult US population is afflicted by knee OA.^13^ Other disorders of the knee joint include meniscus tears, common in young athletes,^14^ resulting in the development of OA in the knee; one study showed that 85% of patients with medial meniscus tears also developed OA.^15^ Although epidemiological studies of OA incidence in the TMJ are not extensive, one article indicates evidence of TMJ-OA in 8%–16% of the population.^16^ Temporomandibular disorders (TMDs), an umbrella term to describe a wide variety of TMJ pathologies, include TMJ-OA (also referred to as degenerative joint disease) as well as other ailments, such as disc pathologies and myofascial dysfunction. Joint disorders also involve changes to muscles, ligaments, tendons, and bones; this review will be focused primarily on the cartilages of the knee joint and the TMJ.

Although the joints have similar anatomy and OA incidence, the knee and TMJ fields display stark differences in primary research, funding, cell-based products in development, and total joint replacement procedures (Table 1). Compared to the TMJ, the knee has a greater quantity of basic and translational research, resulting in more product development and marketed treatments. For example, the knee has a 5.5-fold higher amount of R01 research project grants from the National Institutes of Health (NIH) compared to the TMJ in 2019. The NIH is part of the US Department of Health and Human Services and is the largest biomedical research agency in the world. It is broken down into 27 institutes and centers, which fund scientific grants. There is also a major difference in the amount of end-stage surgical procedures performed on knee and TMJ patients; the knee has approximately a 2,000-fold higher frequency of total joint replacements compared to the TMJ. The dearth of TMJ research presents a pressing challenge toward developing novel cartilage therapies, but by bolstering primary and translational research of the TMJ, new products for its cartilages may be developed.^17^ The lack of translational advancement for the TMJ represents a chokepoint in the development of safe and effective therapeutics for people afflicted by TMDs, which, according to the TMJ Association, totals over 35 million adults in the US.^18^ In this review, we compare the pathologies, anatomical challenges, clinical practices, and products for the cartilages of the two joints within the US medical system and call for improved treatment options for specific TMJ indications. By using the knee orthopedics field as a template to follow in translational pathways, TMJ experts can drive the implementation of new cartilage therapies for millions of TMD patients.

**Table 1:** Comparison of the knee and TMJ fields

	Knee	TMJ
Osteoarthritis incidence	~14%^13^	8%–16%^16^
Professional membership	AAOS membership: 39,195^19^	AAOMS membership: 11,436^20^
PubMed articles^a^	1,852 (in 2019)	288 (in 2019)
R01s (research project grant)^b^	33 (in 2019)	6 (in 2019)
R21s (exploratory/developmental research project grant)^b^	9 (in 2019)	1 (in 2019)
Cell-based therapeutics in development or clinical trials (worldwide)^c^	18^21^	1^d^
Projected number of total joint replacement procedures in 2020 (US only)	882,000–1,783,000^22^ (range of 2020 projections from a variety of historical data)	709^23^ (2020 projection from historic data from 2005 to 2014)

Despite similar incidence of osteoarthritis, the TMJ lags behind in research output, grant funding, cell-based products, and practicing physicians.

a PubMed was searched using the following keyword schemes: ([([tibiofemoral] OR [knee]) AND ([cartilage] OR [meniscus])]) and ([([temporomandibular] OR [jaw]) AND ([cartilage] OR [meniscus] OR [disc] OR [disk])])

b NIH RePORTER was searched using the following keyword schemes limited to project abstracts: ([([tibiofemoral] OR [knee]) AND ([cartilage] OR [meniscus])]) and ([([temporomandibular] OR [jaw]) AND ([cartilage] OR [meniscus] OR [disc] OR [disk])])

c Related to treating cartilage, meniscus, and disc pathologies

d Based on searches based in clinicaltrials.gov across all countries

## Pathology

### The TMJ requires improved diagnostic modalities despite similarities in OA progression

Knee and TMJ OA pathologies have several similarities. This disease involves mechanical and biochemical degradation of cartilage, subchondral bone, and synovium.^24^ Pain is the most common OA symptom, which can range from barely noticeable to severe and debilitating.^25^ Other symptoms include joint stiffness, reduced function or range of motion, and, with severe TMJ-OA, changes in occlusion. Knee OA is traditionally diagnosed with radiography, and early damage can be detected using magnetic resonance imaging (MRI).^26^ For TMJ-OA, panoramic radiography has a low sensitivity for diagnosis.^27^ Cone beam computed tomography (CBCT), a widely used imaging modality, is used as a more reliable diagnostic technique,^28^ and MRI can be used to assess different signs of dysfunction.^29^ Dynamic MRI scoring algorithms, similar to those used to evaluate cardiac wall motion,^30^ can be used for the TMJ, for example, to assess the causes of reduced joint mobility. Reduced mobility is often caused by a displaced disc,^31^ but capsule and ligament pathology may also play a role; these are not routinely examined with imaging. An unusual caveat with TMJ imaging is that radiographic signs alone may not be associated with pain; one study performed CBCT imaging on TMJs of healthy adults with no TMJ complaints, and nearly 40% of the TMJs showed degenerative changes.^32^ Conversely, TMJ pain may not be associated with radiographic signs of disease.^33^ For determining the source of pain in the TMJ, positron emission tomography paired with a computed tomography scan (PET/CT) is being used to image inflammation and bone changes in the TMJ and may be useful for diagnosing TMJ-OA.^34^ Although knee OA is similar in nature to TMJ-OA, additional assessment tools are needed to improve the accuracy of OA diagnosis correlating with symptoms in the TMJ,^35^ thus improving indications for TMJ repair.

### TMJ disorders are more prevalent in women

Women experience higher levels of knee pain than men,^36^ with about a 1.6-fold higher incidence of knee OA.^37^ This difference is likely caused by biomechanical, hormonal, and neural differences,^38^ but a better understanding is needed. Epidemiological studies on gender differences in TMJ-OA are not extensive, but the higher prevalence of TMDs in women has been widely documented; TMDs are up to four times more prevalent in women than men, with women presenting more severe symptoms.^39^ There is evidence of increased amounts of hormone receptors in the TMJ discs of women with TMDs,^40^ but there is conflicting literature showing relationships between TMD prevalence and estrogen levels.^41^ This coincides with a high proportion of young TMD patients compared to knee OA patients.^42^^,^^43^ An earlier onset of TMJ-OA challenges the “overuse phenomenon”—that OA occurs when functional demands exceed the adaptive capacity of the cartilage.^44^ One stark example of age and gender bias in TMDs is the incidence of idiopathic condylar resorption of the TMJ. This disease occurs nine times more frequently in women than men and rarely develops after the age of 20.^45^ In these young patients, TMDs may profoundly affect facial growth, occlusion, and airway dimensions.^46^ Given the severe, unexplained gender discrepancy in the TMJ, which has been called the “TMJ gender paradox,”^47^ deeper understanding of what drives the higher TMD occurrence in young women remains a major milestone for the field.

### The anatomical challenge of sensory structures near the TMJ versus the knee

A major difference between the joints is the location relative to vital structures, which affects joint symptoms, treatment effectiveness, surgical approaches, and adverse events. In the knee, the tibial, peroneal, and saphenous nerves are near the joint (Figure 1), but nerve damage is rare during knee surgery. The TMJ is near multiple important sensory nerves, parts of the inner ear, and the brain (Figure 1). The TMJ’s sensory nerves innervate surrounding masticatory muscles, and spasms in these muscles might be associated with TMJ pain.^48^ One study on 501 TMD patients showed that 60 also had trigeminal neuritis, a condition causing severe, chronic pain.^49^ TMJ disc displacement may compress the mandibular nerve, causing neuropathic pain.^50^ People with TMDs are more likely to have severe tinnitus and vertigo, potentially due to the TMJ’s proximity to the inner ear.^51^ The complex anatomy and associated symptoms can complicate diagnosis and make treatment difficult,^52^ and the outcome of patients with neural and joint symptoms is inconsistent, often resulting in unsuccessful treatments.^53^ As shown in Figure 1, a 3-cm sphere centered on the TMJ disc contains major nerve structures, the inner ear, and the brain, although the same sphere centered on the knee meniscus contains no major sensory structures. In addition to the aforementioned lack of diagnostic modalities, a major hurdle in performing surgery is the TMJ’s close proximity to the brain,^54^ illustrating the anatomical challenge of diagnosing and treating cartilage disorders of the TMJ compared to the knee.

## Current clinical practices

### Divergence of end-stage treatment strategies for OA of the knee and TMJ

**Figure 2:**
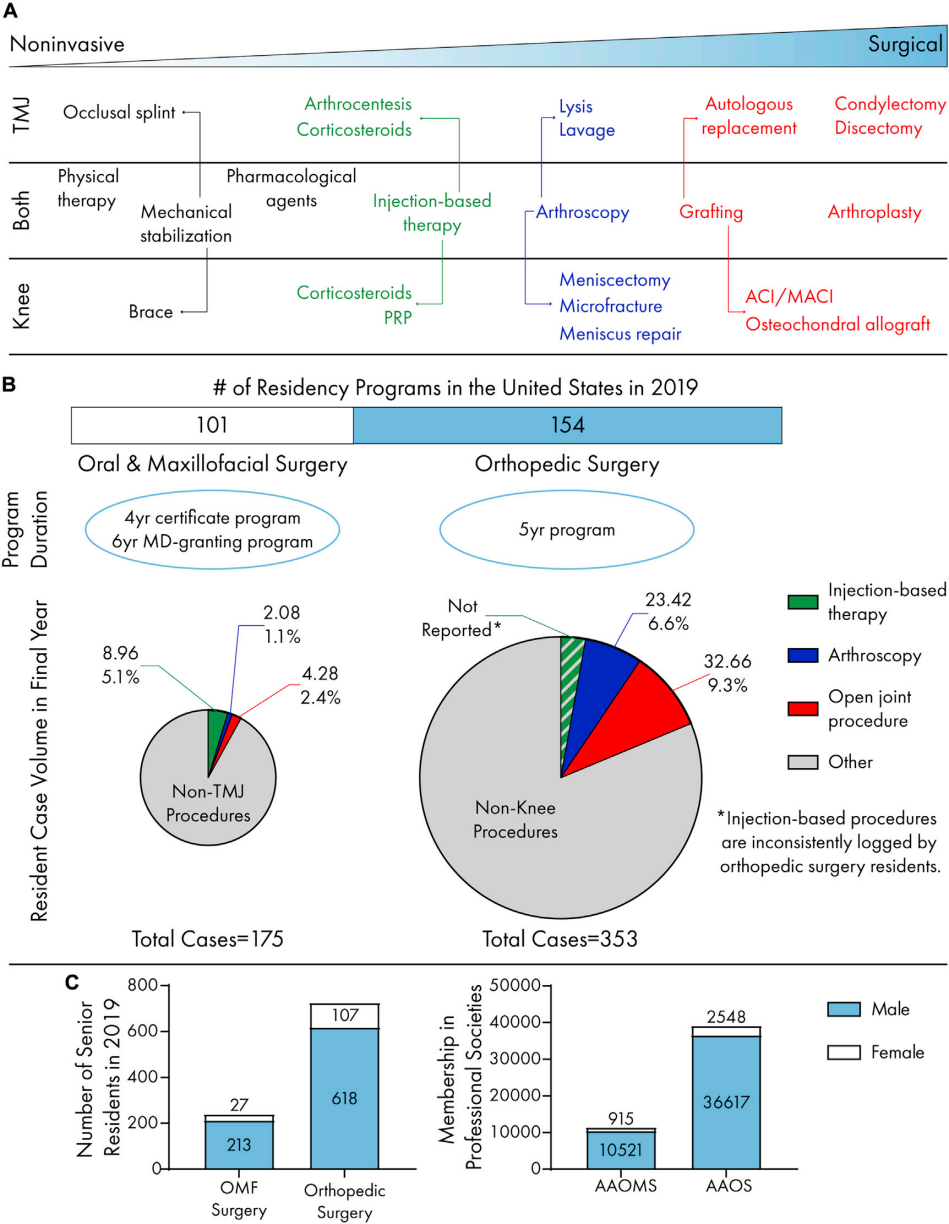
Clinical practices for the knee and TMJ (A) The cartilages of knee and TMJ share similar treatment pathways. Progressing from noninvasive to surgical approaches, conservative treatment is often indicated prior to end-stage surgeries, such as grafting (e.g., fat and rib) for the TMJ and osteochondral allografts for the knee. (B) Orthopedic surgery leads oral and maxillofacial surgery in residency program quantity. Oral and maxillofacial surgery residents are exposed to a lower quantity and percentage of total cases in open joint and arthroscopic procedures compared to orthopedic residents. (C) Males are overrepresented in both senior resident numbers and professional society memberships in AAOMS and AAOS.

The treatment strategies for knee and TMJ cartilage pathologies are similar at first glance (Figure 2A). Both the American Academy of Orthopaedic Surgeons (AAOS) and the American Association of Oral and Maxillofacial Surgeons (AAOMS) list physical therapy, analgesics, and mechanical stabilizers as conservative treatment options for OA.^55^^,^^56^ These relatively noninvasive, early-stage therapies are often undertaken by a variety of providers, such as physicians and physical therapists or, for the TMJ, dentists and dental specialists. If such therapies prove ineffective, frequently, orthopedic and oral and maxillofacial (OMF) surgeons employ injection-based therapies.^57^^,^^58^ There are greater differences between surgical treatment options. Few late-stage TMD patients are referred to an OMF surgeon, indicated by the small number of TMJ surgeries performed. Only 5% of TMD patients are considered candidates for surgery,^59^ despite a lack of positive outcomes with non-surgical approaches. This is reflected in the decline of TMJ surgeries; the number of TMJ arthroscopic surgeries has steadily decreased since the 1990s.^60^ This is in contrast to total knee arthroplasties, which are projected to increase by up to 800% by 2050.^61^ The declining trend in the TMJ field may be attributed to a number of causes, such as disagreement over the suitability or efficacy of surgical approaches.^60^ Conversely, knee cartilages have well-defined treatment algorithms. For example, focal defects are treatable with widely accepted surgical techniques.^21^ In the US, current procedural terminology (CPT) codes are used to report medical or dental services provided by a physician to insurance companies for reimbursement or payment. A higher specificity of knee treatments is shown in a higher quantity of CPT codes compared to the TMJ. For example, TMJ arthroplasty has only three CPT codes although knee arthroplasty has ten.^62^^,^^63^ Knee treatment is covered by medical insurance, and TMDs can be covered under either medical or dental insurance. It is clear that TMD patients need greater access to effective, end-stage treatments with indications that are well vetted among OMF surgeons with specialized training in the TMJ.

### Training disparities between knee and TMJ surgeons

Quality surgical treatment is directly related to physician training. OMF and orthopedic surgery training consists of 4 years of dental or medical school followed by residency (Figure 2B). OMF surgery residency lengths and degree requirements vary significantly across countries; this is reviewed elsewhere.^64^ In the US, OMF surgery residency may either be a 4-year program (single degree) or an MD-granting 6-year program (dual degree). Dual-degree programs make up to 46% of the residency programs.^65^ One measure of surgical training is resident case log volume throughout the duration of the program (Figure 2B). Regardless of residency duration, the Commission on Dental Accreditation (CODA) requires that OMF residents log 175 major procedures in their final year.^66^ A survey of senior OMF residents in the US showed that only ∼3.5% of these cases involve TMJ arthroscopic or open joint procedures.^67^ Most TMJ-specific treatments performed by residents were injection based.^67^ At programs lacking OMF residencies, TMDs may be covered by plastic surgery or otorhinolaryngology residencies, but TMDs are not a focus. In general, orthopedic surgery residents log 200–600 cases in the final year,^68^^,^^69^ and in 2019, knee-specific arthroscopy and open joint surgery accounted for ∼16%.^70^ Following their 5-year residency, most orthopedic surgeons complete a fellowship year,^71^ and those interested in knee cartilage typically choose a sports medicine or arthroplasty fellowship. Although the CODA-accredited endoscopic maxillofacial fellowship provides more substantial TMJ-related training,^72^ fellowships are not as popular among OMF surgeons.^73^ If better end-stage treatment options are to become available, there will be a greater need for TMJ specialization to meet the surgical needs of TMD patients.

### From incidence to clinic: gender imbalances in physician populations and clinical trials

Gender imbalances are present in the demographics of knee and TMJ patients and the surgeons that treat them, and these disparities have a clinical impact on the treatment of knee- and TMJ-related ailments. Because there are significantly more women who experience OA and TMDs, it is important to account for gender-based differences in treatment development. For example, women report more pain before and after total knee arthroplasty when compared to men.^74^ Similarly, the percentage of women reporting orofacial pain at routine dental visits was triple that of men.^75^ Although some have focused on psychosocial factors to explain this difference,^76^, ^77^, ^78^ it is important to consider the ample evidence showing that women’s analgesic response is physiologically different from men’s.^79^ Interestingly, a recent review noted that physicians are more likely to recommend greater pain intervention for patients of the same gender.^80^ This is important, given that most knee and TMJ OA patients are female, although orthopedic and OMF surgeons are predominantly male (Figure 2C). In the last decade, approximately half of medical and dental school graduates were women, but the percentage of female residents in both orthopedic and OMF surgery programs has only been about 15%.^81^^,^^82^ This imbalance is greater within professional societies; women make up only 6.5% and 8.0% of the AAOS and AAOMS membership, respectively.^83^^,^^84^ Reducing this disparity among orthopedic and OMF practitioners will help ensure that gender-based differences are not overlooked in patient treatment.

Potential therapeutics must go through preclinical and clinical trials to gain regulatory approval. Potential product sponsors must consider careful design of such trials, specifically, the gender of the participants. With a vast majority of people experiencing TMDs being women, the NIH’s policy on “sex as a biological variable” is particularly relevant.^85^^,^^86^ This strongly encourages researchers to consider gender-based differences throughout the translational process. Unfortunately, meta-analytic studies have shown that gender differences in clinical trials are underreported.^87^ Translational scientists must consider how gender disparities in knee and TMJ OA patients may not only affect the demographics of clinical trials but, subsequently, the landscape of commercial products.

## Commercial products

### Joint prostheses: Successes in the knee and catastrophic setbacks in the TMJ

Partial or total joint replacement is the current solution for patients with cartilage pathologies that fail to improve with less-invasive treatments. The first hemiarthroplastic knee device, a tibial plateau prosthesis, was designed by McKeever in 1957.^88^^,^^89^ In 1963, Christensen published on a fossa-eminence prosthesis for TMJ hemiarthroplasty.^90^ These devices paved the way for the development of total joint implants in the US. The early total knee devices include the Freeman-Swanson knee and the Geomedic knee.^89^ In the 1970s and ‘80s, the Oxford knee and the New Jersey low-contact-stress knee significantly improved mobility and are still used.^91^ Today, there are over a dozen knee replacement manufacturers collectively offering a wide range of customization options.^91^^,^^92^

Unlike the knee, TMJ prosthesis development has been slow and controversial. In 1983, the US Food and Drug Administration (FDA), the regulatory body responsible for determining safety and efficacy of therapeutics, cleared Vitek’s Proplast-Teflon implant for TMJ disc replacement, despite evidence of fragmentation with similar Teflon-based hip replacements.^93^ Given the joint’s proximity to the brain, the implant’s failure led to catastrophic outcomes, such as particulate migration and cranial breaching.^94^^,^^95^ By 1990, the FDA rescinded clearance of the implant and issued a recall a year later.^96^ In 1993, the FDA reclassified all TMJ prostheses as class III, a designation reserved for devices posing the greatest risk,^97^ requiring more rigorous premarket approval and stifling production of all TMJ implants. Currently, there are only four FDA-approved TMJ implants.^98^ Nexus makes both a partial and total metallic joint, and Zimmer-Biomet and TMJ Concepts make metal/polymer total joint replacements.^93^ This is in stark contrast to the hundreds of total joint replacement systems available for the knee.^89^

### The TMJ field trails the progress of tissue engineering in the knee

An important measure of an implant’s success is its long-term performance and lifetime. Approximately 82% of total knee replacements survive 25 years.^99^ Although most surveillance studies for TMJ implants are ongoing, Zimmer-Biomet reports that implant survival is 86% after 10 years.^100^ The average age of a TMJ-implant recipient is only 34.9 years compared to 67.5 years for knee implant recipients.^43^^,^^101^ Assuming an implant lifetime of 20 years and an average life expectancy of 78.6 years in the US,^102^ a TMJ patient is likely to need two revision surgeries; a knee patient is unlikely to need any. This disparity underscores the TMD patient’s dire need for high-quality, long-lasting replacement options for treatment of late-stage, degenerative TMDs. Tissue engineering offers a promising long-term alternative to alloplastic implants, thus potentially eliminating the need for revision surgeries.

In 2016, the FDA approved matrix-induced autologous chondrocyte implantation (MACI), a two-surgery process utilizing expanded cells seeded on a porcine collagen membrane, for the repair of knee cartilage defects.^103^ Several more tissue-engineered products, such as NOVOCART 3D for articular cartilage and Chondrogen for the meniscus, are currently proceeding through the regulatory pipeline.^21^ Some tissue-engineered products employ an allogeneic approach; one example, Revaflex, reports encouraging results in clinical trials.^21^^,^^104^ Given this established precedent, continued development of tissue-engineered products for the knee cartilages will proceed. Development of cell-based therapeutics for the TMJ cartilages is still in its nascent stage, with only one clinical trial based in Brazil.^105^ Although autologous grafts (e.g., fat and rib) for the TMJ offer a tissue-based option,^106^^,^^107^ there are still no approved, tissue-engineered products for the TMJ cartilages in the US.

## Future directions

### The vicious cycle of translating TMJ research

**Figure 3:**
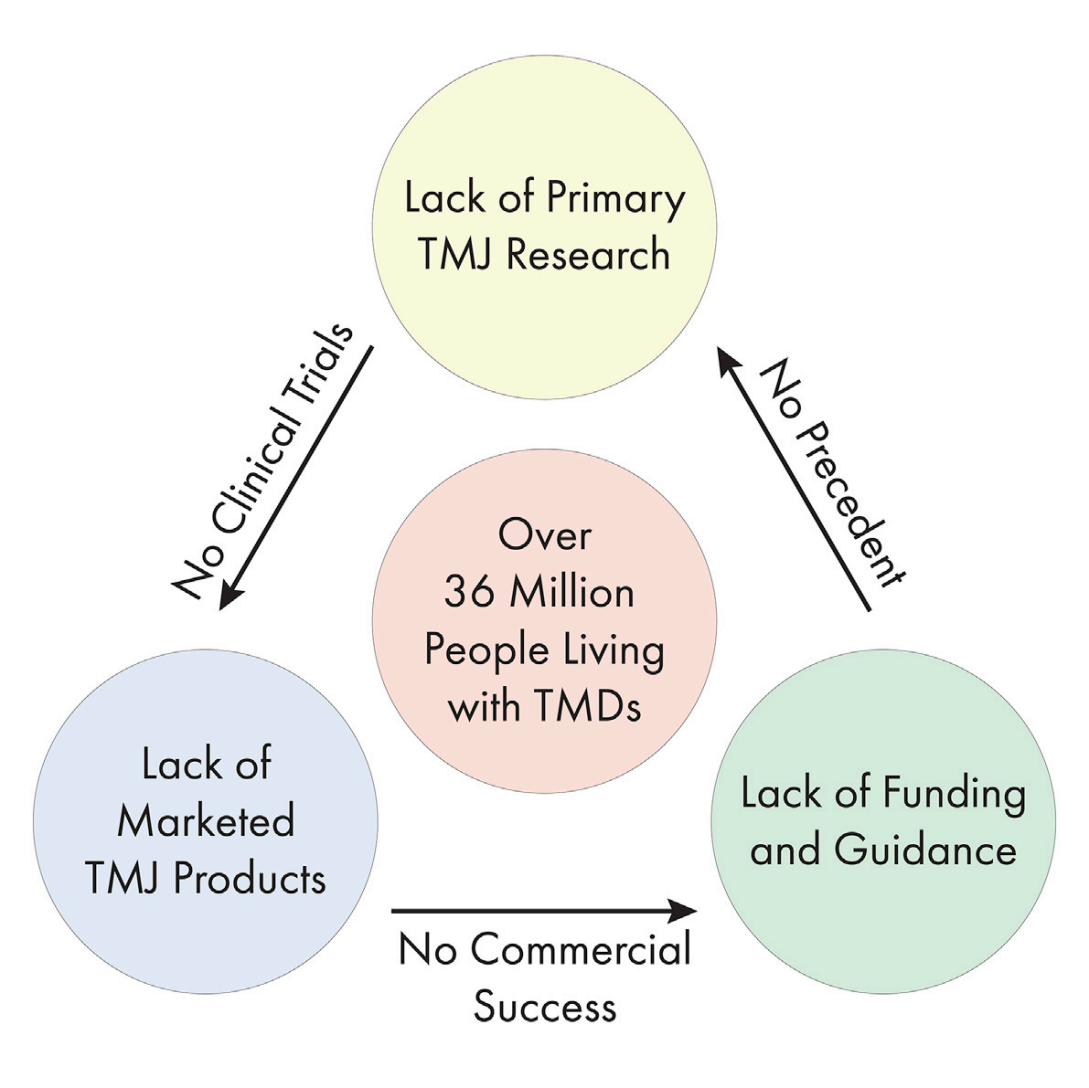
The vicious cycle of TMJ translational research Primary research is lacking in the TMJ field, leading to little translation and resulting human clinical trials. Without clinical trials, approved, marketed products do not exist, resulting in little to no commercial market for TMJ products. This disincentivizes regulatory and funding agencies from publishing guidance and providing funding for the TMJ field, feeding back into the loop and resulting in a lack of precedent for researchers.

Tissue engineering can potentially offer long-term solutions for knee and TMJ cartilage ailments. With multiple products either approved or in trials, tissue engineering toward regeneration of knee cartilages is poised to be a major early success of regenerative medicine, acting as a template for other joints, such as the TMJ. Although the knee and TMJ fields had similar start points in the 1950s and ‘60s with joint replacement devices, the catastrophic failure of the Proplast-Teflon protheses affects the TMJ field to this day. The knee field enjoys much success in terms of quantity of research output, funding, marketed products, and regulatory guidance, although the TMJ field is relatively stagnant in nearly all areas. Low research output, especially in translational science, leads to fewer innovative therapeutics that make it to clinical trials. The resulting dearth of products for TMJ cartilages limits commercial success, disincentivizing financial and regulatory support, feeding back into the vicious cycle as a lack of precedent (Figure 3). By increasing research output, bolstering training opportunities, narrowing and specifying indications for TMJ cartilages, establishing a commercial TMJ landscape, and publishing guidance documents, the field can accelerate translational research to break the vicious cycle.

### Increasing the quantity of rigorous TMJ research

There is a critical need for increased basic and translational research output to energize the TMJ field. Within the US, the number of TMJ-related, NIH-funded grants drastically trails those of the knee (Table 1), illustrating the need to further mature the TMJ field. In a recent report, the National Academies of Science, Engineering, and Medicine recommended bolstering different aspects of TMJ research.^108^ For example, the report calls for the creation of a national collaborative research consortium and expansion of TMJ research. For orthopedics, the Orthopedic Research Society (ORS) helps facilitate these activities through their annual meeting. This meeting brings together orthopedic surgeons, biologists, engineers, and other experts across various fields. This fosters a collaborative environment for those within orthopedics, both researchers and practitioners, to discuss interdisciplinary research and form collaborations. Although the TMJ Bioengineering Conference occurs every 2 years and achieves the same goal as the ORS annual meeting on a smaller scale, the TMJ field is more fragmented. There are multiple different symposiums, such as the meetings of the American Society of TMJ Surgeons, the AAOMS, the American College of Oral and Maxillofacial Surgeons, and the American Academy of CranioMaxillofacial Surgeons, that discuss the TMJ, but the field does not have one meeting that brings together experts from different fields at the same scale of ORS. One can consider a TMJ meeting, modeled after ORS, where researchers and clinicians meet to discuss current treatment issues and how interdisciplinary approaches can be developed toward relieving TMJ ailments. Additionally, ORS may consider cosponsoring a TMJ-specific meeting to bring together researchers, orthopedic surgeons, and OMF surgeons to discuss how treatments and approaches in the knee cartilages could be transferred to the TMJ. By using orthopedic meetings as a template, the TMJ field can accelerate the basic and translational research toward clinical trials to develop effective TMJ therapeutics.

Without a large quantity of interdisciplinary research, the TMJ field will not be able to establish a base to propel itself forward. One of the major issues is that TMDs disproportionately affect up to 17% of all American women.^18^^,^^39^ Understanding fundamental science will shape the approach of translational research, such as in the design of preclinical animal studies. Although basic science and translational research have already resulted in a steady rise of TMJ-related publications since 2006,^109^ federal bodies, such as the National Institute of Dental and Craniofacial Research (NIDCR), NIH, and FDA, still push for increased research.^108^ This will not only lead to increased output but also maintain a high standard of scientific rigor, as those bodies require that grant applications and manuscripts undergo peer review. On average, an R01 grant from the NIH leads to 7.36 published research articles with almost 300 citations.^110^ Similar to ORS sponsoring a combined meeting, the NIDCR and institutes such as the National Institute of Arthritis and Musculoskeletal and Skin Diseases (NIAMS), two US NIH institutes that the TMJ and knee commonly fall under, may consider publishing dual requests for applications that study the knee and TMJ cartilages under one grant. These could focus on transferring the knee knowledge, equipment, and protocols to the cartilages of the TMJ, providing incentive for established musculoskeletal researchers to extend a branch into the TMJ field. By increasing grant funding through various channels, such as the NIH, TMJ research output will be closer to the level of the knee field.

### Bolstering training opportunities for researchers and physicians

As research output rises, more trained researchers and surgeons will be needed. The NIH F-series of grants is targeted toward students and postdoctoral fellows to support both individuals (e.g., salary, stipend, and tuition) as well as their proposed research. The funding success rates of the NIDCR grants for pre- (F31) and post-doctoral (F32) candidates were 70% and 35% in 2019, respectively.^111^ Considering both of these success rates trend in the top third of all NIH institutes,^111^ the NIDCR is commended for their commitment to funding junior researchers. However, total funds disbursed by the NIDCR ranked in the bottom half of all NIH institutes.^111^ Compared to the NIDCR, the NIAMS F31 and F32 success rates were lower in 2019, about 18% for both mechanisms.^111^ Although the number of candidates funded were similar for each mechanism, the total number of applicants for NIAMS fellowships was drastically larger than those of the NIDCR;^111^ thus, the NIDCR could improve their outreach and advertising of such mechanisms. A mechanism to bridge postdoctoral scientists to their early career (e.g., junior faculty) is the K99/R00 grant, which includes both a mentored and independent research phase. In 2019, the K99 application numbers were comparable between the NIDCR and NIAMS, but the NIDCR maintained a higher success rate of 36.8% compared to 20.0%.^111^ Clearly, the NIDCR is committed to developing researchers’ careers in the craniofacial, dental, and TMJ fields. By maintaining high success rates, increasing funding disbursed, and bolstering outreach efforts for these awards, the NIDCR will increase the number of trainees pursuing a TMJ-related career.

As discussed above, orthopedic surgery residents gain exceptional experience in open joint surgeries and arthroscopy of the knee (∼16% of cases)^70^ compared to the equivalent for the TMJ (∼3.5%) for OMF residents.^67^ Development of reliable diagnostic modalities and clear treatment algorithms to address specific pathology will increase TMJ surgery case volume for OMF residents, thus better equipping future surgeons for implementing new therapeutics. An additional option would be to develop a TMJ-specific surgical fellowship for OMF residents modeled after fellowships for orthopedic surgeons. Lastly, as the field matures, the need for dual-degree clinician-researchers will grow, which is expected to spur the development of novel therapeutics. The dual-degree F30 fellowship success rate for the NIDCR in 2019 was in excess of 80%,^111^ indicating that the institute is committed to developing clinician-researchers and their ideas toward therapeutics. Early career medical professionals might bridge their careers into research with a K08 clinical investigator award, which provides these individuals with an intensive research career development experience. In contrast to the F30 mechanism, the K08 success rate for the NIDCR (25.0%) trails that of NIAMS (56.3%), although they maintain relatively similar applicant numbers.^111^ Encouragement of clinician-researchers to apply for funding at the success rate of the F30 award and continuing to increase success rates at the K08 level will grow and sustain the number of TMJ physicians and translation of novel therapeutics.

### Narrowing and specifying indications for TMDs toward establishing the commercial landscape for TMJ cartilage products

As researchers and clinical practitioners continue to grow the field in number and research output, there will be a push to narrow and specify indications for the TMJ-specific conditions amenable to surgical management. Currently, the term TMD encompasses many different conditions, including muscle and joint problems.^112^ This has led to confusion in the field today that causes conflicting paradigms for treating certain indications. Toward solving this, the National Academies’ report recently recommended establishing a national TMD registry to track incidences, indications, and treatment pathways toward establishing best practices.^108^ A template for the TMJ field may be the AAOS American Joint Replacement Registry that has recorded procedural data, post-operative data, and patient-reported outcome measures on over 2,000,000 joint replacement procedures for the knee and hip since 2009.^113^ Continued clinical research output, specifically retrospective and meta-analytic studies on certain indications, will additionally improve the clinical body of knowledge.

Establishing specific indications for TMDs is crucial to a healthy market for the TMJ field; without a clear indication, there is no commercial product. Due to a lack of indications, it is not clear how TMJ tissue-engineered products might be implemented.^114^ By establishing indications, more TMJ scientists and clinicians will attempt to translate technologies, therapeutics, and devices from the benchtop to the bedside. In the knee, MACI is indicated for patients with symptomatic, full-thickness knee cartilage defects who have failed conservative treatments.^115^ The TMJ market would benefit from using MACI and other knee cartilage therapeutics as a template for TMJ tissue-engineered products. As translation occurs for various TMJ therapeutics, additional CPT codes and surgical procedures will likely be needed. Historically, only 5% of TMDs are candidates for surgical intervention.^116^ These few surgical cases only include three CPT codes for TMJ arthroplasty, compared to ten for knee arthroplasty. As therapeutics are developed, specifically those for late-stage pathology, it is likely that the number of CPT codes and surgical TMD cases will increase.^62^^,^^63^ CPT codes for these indications will need to be sufficiently supported by science for third-party reimbursers to support payment for the procedures. Furthermore, additional CPT codes specifying indications for late-stage pathology may further bolster medical insurance support (i.e., as compared to dental insurance). Additionally, following the specificity seen in CPT codes for the knee as a template, progress is also needed in delineating CPT codes for early-stage TMDs to support non-surgical treatment. Finally, specific terminology delineating between the various muscle and joint problems would be a step toward dismantling the umbrella term “TMD.” This would clarify communication among researchers, physicians, and patients, improving granularity in treatment algorithms and garnering support for TMDs for treatment under medical insurance as opposed to dental insurance. A recent example of this is the recommendation provided by the International Research Diagnostic Criteria for Temporomandibular Disorders Consortium Network and Orofacial Pain Special Interest Group, which attempts to delineate among the various myogenous and arthrogenous conditions,^117^ but this report has been deemed to fall short.^112^ Additional work is needed in order to delineate the term further and identify appropriate indications. As more entrepreneurial ventures are established, a larger market for therapeutics indicated for specific, end-stage TMDs will arise, laying the groundwork for the TMJ commercial landscape.

### Implementing industry guidance for treatment of TMJ cartilages

As scientific entrepreneurs establish TMJ startup companies, they will look to regulatory agencies for guidance. The FDA has previously given specific guidance for products intended to repair or replace knee cartilage, with specific recommendations to establish safety and efficacy.^118^ Analogous FDA guidance for the TMJ is necessary if new cartilage products are to emerge; to this end, the knee cartilage guidance document may serve as a template for the TMJ. Specific considerations, such as proximity to crucial sensory structures, the mechanical loading environment of the joint, and appropriate animal models, should be included. Defining regulatory jurisdiction would also be helpful by delineating various types of therapeutics (e.g., drug, biologic, device, or combination product), not only for the TMJ but also the knee. Autologous materials have been used in the past for the TMJ,^17^ but recent animal studies suggest that allogeneic approaches are also safe.^119^ Assays required to show safety for allogeneic approaches compared to autologous approaches may be considered. Due to the dearth of precedent for both the knee and TMJ, the FDA might consider providing early communication and advice through existing designations and programs, such as breakthrough and fast track designations and priority review and accelerated approval programs, as applicable to product sponsors. Additionally, establishing regulatory guidance in the benchtop and preclinical phases of the translational paradigm would be useful. For example, for NIH-funded grants with animal studies, perhaps the FDA and the appropriate NIH institute could collaborate to provide early regulatory advice to the principal investigator. This guidance and support would undoubtedly improve the success of translational TMJ research.

## Conclusions

The nature of translational research is inherently arduous, with many choke points frequently leading to a vicious cycle (Figure 3). However, there are just as many possibilities to break this cycle. As the NIDCR moves forward with funding various projects, they should consider funding TMJ cartilage-specific grants to encourage focused research in the field. Although current support at the R-series level is insufficient (Table 1), early career researchers are well funded through F-series mechanisms, which bodes well for the future of the TMJ field. Increasing funding and outreach for such mechanisms while maintaining success rates would be beneficial to the field. For surgical trainees, bolstering the number of TMJ cases encountered throughout residency, developing TMJ surgical fellowships, and encouraging clinicians to apply for F30/K08 awards will increase the supply of TMJ-specific OMF surgeons to perform technically challenging cartilage procedures and explore the clinical efficacy of new therapeutics. Clarification of indications and treatments for TMDs through a national registry will be of great value to entrepreneurs attempting to translate technologies. By doing so, markets for such technologies will arise so that additional CPT codes specific to cartilage indications will be established, enabling additional insurance coverage for TMDs. Finally, guidance published by the FDA will enable translational studies to support safety and efficacy of TMJ cartilage products, especially tissue-engineered implants. These documents should include specific considerations for TMJ cartilages, such as proximity to crucial structures and timely guidance for nascent products. Similar approaches and suggestions have resulted in the development and translation of tissue-engineered products for knee cartilages and can thrust the TMJ field forward. With knee orthopedics as a template, the TMJ field can make great strides toward ameliorating the symptoms that millions of TMD patients experience on a day-to-day basis, drastically improving their quality of life.
